# Diabetes mellitus and comorbidities – A cross-sectional study with control group based on nationwide ambulatory claims data

**DOI:** 10.25646/8327

**Published:** 2021-06-16

**Authors:** Christian Schmidt, Lukas Reitzle, Rebecca Paprott, Jörg Bätzing, Jakob Holstiege

**Affiliations:** 1 Robert Koch Institute, Berlin, Department of Epidemiology and Health Monitoring; 2 Central Research Institute of Ambulatory Health Care in Germany (Zi)

**Keywords:** DIABETES MELLITUS, AMBULATORY CLAIMS DATA, COMORBIDITY, DIABETES SURVEILLANCE

## Abstract

As a condition, diabetes mellitus is associated with risk factors and diseases such as obesity. At the same time, cardiovascular diseases are a frequent consequence of diabetes. There have yet to be any findings on the Germany-wide prevalence of diabetes and diabetes comorbidities based on statutory health insurance data. This study estimates the documented prevalence of diabetes in 2019 on the basis of all ambulatory physicians’ claims data of German statutory health insurance. In addition, the prevalence of obesity, high blood pressure, coronary heart disease, heart failure, stroke and depression is calculated for diabetes and non-diabetes patients, and the prevalence ratio (PR) is determined as a quotient. The approach used was a case-control design, which assigns a control person without diabetes to each diabetes patient who is similar in terms of age, region and sex.

In diabetes patients, a PR greater than 1 was observed for all examined diseases across all age groups, thus demonstrating a higher prevalence compared to persons without diabetes. The highest PR across all age groups for women (3.8) and men (3.7) was found for obesity. In a comparison over time, documented prevalence figures of diabetes in Germany stagnate. With the exception of depression, the documented prevalences of comorbidities correspond well with the prevalences found in population-wide examination surveys.

## 1. Introduction

Over recent decades, type 2 diabetes has gained in public health relevance both in Germany and worldwide. On the basis of various data sources, studies on the development of diabetes prevalence show a tenfold increase in Europe and Germany since the 1960s [1]. Population-wide results for Germany also show an increase in prevalence over the last two decades, and at the same time, there is evidence of a high potential for diabetes prevention [2, 3]. Advanced age and a family history of the disease, as well as behavioural risk factors such as a lack of physical activity, smoking and poor diet resulting in obesity have been shown to be the main risk factors of type 2 diabetes [4]. In addition, settings-based risk factors for type 2 diabetes are also being discussed. In particular, living environments with few opportunities for physical activity, an oversupply of energy-rich food or living in a neighbourhood where many people have formally low levels of education have been studied as settings-based risk factors [5, 6]. In contrast to type 2 diabetes, the other types of diabetes, type 1 and type 3 diabetes, are relatively rare and have other causes.

Long-term elevated blood glucose levels in people with diabetes damage the small blood vessels (microangiopathy) and nerves (diabetic polyneuropathy) and can typically lead to secondary diseases of the kidneys, eyes or feet [7]. In addition, diabetes is an independent risk factor, specifically for cardiovascular diseases such as coronary heart disease (CHD) and stroke [8].

For Germany, survey data from the Robert Koch Institute (RKI) for persons aged 50 and older show that the age-and sex-adjusted odds of having high blood pressure or a cardiovascular disease is 3.60 and 2.35 times higher respectively in persons with diabetes compared to persons without diabetes [9]. In addition, diabetes patients are also more likely to suffer mental disorders and, in particular, depressive disorders [10]. Irrespective of whether a disease such as CHD is to be regarded as a secondary disease of diabetes or, like depression, as a common concomitant disease, the simultaneous presence of at least one other additional disease is referred to as comorbidity.

Increasingly, statutory health insurance (SHI) claims data are being used to assess the frequency of common diseases such as diabetes [11–17]. There are also occasional analyses of claims data on the frequency of risk factors or comorbidities in persons with diabetes compared with persons without diabetes in insurants of single SHI funds. As these analyses are based on data from a single health insurance fund [18, 19], their results are not, however, readily transferable to the totality of all persons insured by SHI [20].

In diabetes surveillance at the RKI, in addition to diabetes prevalence, relevant risk factors as well as secondary and concomitant diseases of diabetes are presented and recurrently reported [21]. Data from the population representative RKI surveys and claims data are used to populate indicators [22]. This study aims to examine the prevalence of diabetes by age and sex and the frequency of secondary and concomitant diseases based on 2019 Germany-wide SHI claims data. The selection of diseases is based on an expert-consented list that was developed within the framework of diabetes surveillance. The data basis of the analysis is the full sample of ambulatory claims data for the year 2019 [23]. A cross-sectional study was conducted with a control group in order to compare prevalence values between persons with and without diabetes. In addition to age group-specific observations, the focus is on comparing the sex-related outcomes of diabetes comorbidity burdens. The study results are compared with those of the German Health Interview and Examination Survey for Adults (DEGS1) [24], which was conducted between 2008 and 2011 as an interview and examination survey by the RKI.

## 2. Methodology

### 2.1 Study data

The analysis was based on the pseudonymised Germany-wide ambulatory claims data from all health insurance funds in accordance with article 295 of the German Social Code (SGB) V for 2019 for all patients with SHI, provided they had at least one encounter in ambulatory care in the year of study. In total, the 2019 data contain information on the ambulatory SHI-accredited physician medical care provided to 56,648,639 patients of adult age. In addition to sociodemographic data, e.g. on a patient’s age, sex and district of residence, these data also include information on the billed ambulatory medical services and diagnoses, type of physician, e.g. specialists, or the regional Association of Statutory Health Insurance Physicians at which the practice is licensed by the SHI. The data are kept at the Central Research Institute of Ambulatory Health Care in Germany (Zi). To avoid the re-identification of individuals, all information was transmitted in aggregated form and with a minimum of 30 persons per group set.

**Info box Diabetes comorbidities: Case definition and description**

**Obesity**

**ICD-10 codes:** E66.–

► Obesity due to excessive calorie intake, obesity due to medication and other or unspecified forms of obesity

**High blood pressure**

**ICD-10 codes:** I10.–, I11.–, I12.–, I13.–, I15.–

► Essential hypertension, secondary hypertension and diseases of the heart or kidney caused by hypertension

**Coronary heart disease (CHD)**

**ICD-10 codes:** I20.–, I21.–, I22.–, I23.–, I24.–, I25.–

► Angina pectoris, heart attack and chronic ischaemic heart disease

**Heart failure**

**ICD-10 codes:** I50.–, I11.–, I13.0, I13.2

► Heart failure, also as a result of high blood pressure

**Stroke**

**ICD-10 codes:** I63.–, I64.–, I69.3.–, I69.4.–

► Cerebral infarction, stroke and their consequences

**Depression**

**ICD-10 codes:** F32.–, F33.–, F34.1.–

► Depressive disorders and long-lasting depressive mood

### 2.2 Definition of diabetes and its comorbidities

The definition of diabetes was based on the code provided by the 10th revision of the International Statistical Classification of Diseases and Related Health Problems (ICD-10) used in billing claims data. Insured persons who were diagnosed with diabetes mellitus (ICD-10: E10–E14) documented as confirmed in at least two quarters of 2019 (M2Q criterion) were counted as having diabetes in accordance with existing definitions [15] and recommendations [25, 26]. The selection of diagnoses for the definition of comorbidities (Info box) was made according to existing case definitions used for the analyses of claims data [11–14] and the M2Q criterion was applied throughout.

The documented prevalence of diabetes in 2019 was calculated as the number of persons with diabetes as a percentage of the total population of SHI-insured persons as of 1 July 2019 according to the official statutory health insurance member statistics (KM 6 statistics) [27] across the age groups 18 to 29, 30 to 59, 60 to 79 and 80 and older. The KM 6 statistics provide the absolute numbers of insured persons in the lower age segment exclusively for the age groups 0 to 14 years and 15 to 19 years. Since the absolute number of insured persons aged 18 and 19 cannot be taken directly from the KM 6 statistics, this figure was estimated as a basis for determining the size of the insured population 18 years and older in 2019. This was done under the assumption that the distribution of the number of insured persons by age within the 15 to 19 age group corresponded to that of the 2019 German population figures for this age group.

### 2.3 Sample design and study implementation

To compare the prevalence of cardiovascular diseases, high blood pressure, depression and obesity in people with and without diabetes in 2019, the study applied a case-control design. The design randomly assigned each insured person with diabetes (case) to an insured person as a control who had encounters in ambulatory care in at least two quarters in 2019 and did not have a documented diabetes diagnosis in 2019 or in any previous years. Matching was done by age group (5-year age groups), sex and place of residence (17 regions representing the different Associations of SHI-accredited physicians in Germany in order to control for the occurrence of these known influencing factors between the study groups.

In addition to prevalence estimates of comorbidities in both groups, relative differences between groups were assessed by the prevalence ratios (PR) which were calculated as the ratio between the prevalence in the group with diabetes and the prevalence in the group without diabetes. The prevalence in persons with diabetes and persons without diabetes as well as the calculated PR value are presented for each disease according to age group (18-to 29-year-olds, 30- to 59-year-olds, 60- to 79-year-olds, and 80-year-olds and older) for the total estimator and separately for women and men.

Data extraction and analysis were carried out using SAS 9.4 software and results visualised with the freely avail able R version 3.6.1 program using the tidyverse program package [29].

## 3. Results

### 3.1 Sociodemographic factors and health care use

Table 1 provides an overview of sociodemographic factors and SHI-accredited physician appointments and compares the two groups studied. In total, more than seven million people with diabetes, as per the definition provided, were identified and compared with an equal number of controls. Based on the study design, the proportion of women was the same in both groups (49.79%). The age in the two groups showed an almost identical mean (cases: 68.99 years, controls: 68.93 years) and only slight differences in the mean spread (standard deviation; cases: 13.71, controls: 13.84). The utilisation of ambulatory services and the number of treatment cases was considerably higher in diabetics than in controls (Table 1).

### 3.2 Prevalence of documented diabetes

**Figure 1:**
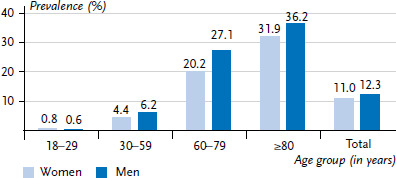
Documented prevalence of diabetes by age group and sex in 2019 (n = 3,518,968 women, n = 3,549,281 men) Source: Germany-wide claims data from SHI-accredited physicians for adults covered by statutory health insurance, own calculations

Figure 1 shows the prevalence of diabetes according to claims data for the year 2019 across the four age groups as well as the overall estimate for persons aged 18 and older (shown separately for women and men). Overall, the prevalence of diabetes increases considerably with age. Whereas the documented diabetes prevalence in women is 4.4% in the 30 to 59 age group, this rises to 20.2% in the 60 to 79 age group and is 31.9% in the age group 80 years and older. In men, the prevalence is 6.2%, 27.1% and 36.2% for the three age groups mentioned. Only in the 18 to 29 age group is the prevalence higher for women (0.76%) than men (0.64%). Across all age groups, prevalence for women is lower (11.0%) compared to men (12.3%).

### 3.3 Prevalence and prevalence ratios of diabetes comorbidities

**Figure 2:**
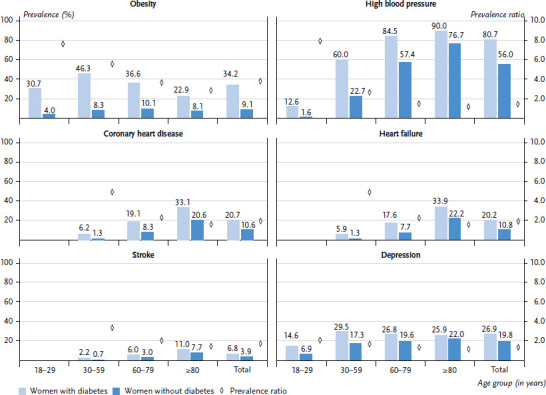
Documented prevalence and prevalence ratio for selected diseases in women with and without diabetes by age group (n = 3,518,968 women with diabetes, n = 3,518,968 women without diabetes) Source: Germany-wide claims data from SHI-accredited physicians for adults covered by statutory health insurance, own calculations

**Figure 3:**
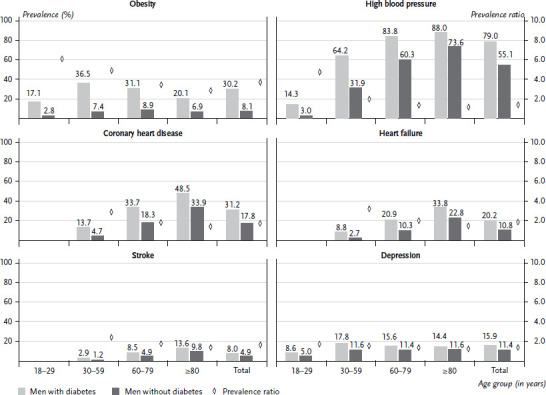
Documented prevalence and prevalence ratio for selected diseases in men with and without diabetes by age group (n = 3,548,968 men with diabetes, n = 3,548,968 men without diabetes) Source: Germany-wide claims data from SHI-accredited physicians for adults covered by statutory health insurance, own calculations

Figure 2 and Figure 3 show prevalence estimates of comorbidities in women and men with diabetes and without diabetes. A PR greater than 1 indicates that the prevalence of the respective condition is higher in the group with diabetes than in the group without diabetes. No values are shown in cells where the number of persons included in either study group is less than 30. This applies to the 18 to 29 age group for heart failure, CHD and stroke in women and men.

### 3.4 Results by age group for women

Across all age groups, obesity prevalence (34.2%) is 3.8 times higher in the group of women with diabetes compared to those without documented diabetes (9.1%). Hence, of all the diseases analysed here, obesity has the strongest association with diabetes in women. In the age group of 18- to 29-year-old women with diabetes, the prevalence of obesity is 7.6 times higher (30.7%) compared to those without diabetes (4.0%), the highest relative difference in prevalence between the two groups studied. The PR for obesity decreases with age: it is still 5.6 in the 30 to 59 age group, falling to 3.6 in the 60 to 79 age group and 2.8 in the age group 80 years and older. The decrease in PR across age groups is due to the prevalence of obesity in women, with diabetes decreasing earlier and more with age. While the highest prevalence of obesity (46.3%) among women with diabetes is found in the 30 to 59 age group, the highest prevalence among women without diabetes (10.1%) is found in the 60 to 79 age group. In this age group, the prevalence for women with diabetes is 36.6% and thus already considerably lower compared to younger age groups.

Across all age groups, the prevalence of hypertension in women with diabetes is 80.7%, 1.4 times higher than in women without diabetes (56.0%). Women with diabetes are eight times more likely to have documented high blood pressure in the youngest age group of 18- to 29-year-olds, with a prevalence of 12.6%, than women without diabetes (1.6%). With increasing age, the differences in prevalence between the study groups decrease, a fact due to a higher relative increase in prevalence in the group of women without diabetes. In the group of women aged 80 years and older, the prevalence in the group with diabetes is 1.2 times higher at 90.0% compared to the group without diabetes (76.7%). A similar picture can also be seen for heart failure, stroke and CHD. All these cardiovascular diseases show large relative differences in prevalence between the study groups, especially in the young age groups (30- to 59-year-olds), which decrease with rising age. Across all age groups, women with diabetes show a 1.7- to 1.9-fold higher prevalence for heart failure (20.2%), CHD (20.7%) and stroke (6.8%) compared to women without diabetes.

Across all age groups, the prevalence of depression is around 1.4 times higher in women with diabetes than in women without diabetes (26.9% vs. 19.8%). Here, too, women with diabetes in the youngest age group of 18- to 29-year-olds show the highest relative difference, with a PR of 2.1, compared to women without diabetes (6.9%). Depression prevalence (29.5%) is highest among women with diabetes in the 30 to 59 age group, whereas the highest prevalence in women without diabetes (22.9%) is found in the age group 80 years and older.

### 3.5 Results by age group for men

Obesity prevalence among men with documented diabetes (30.2%) is 3.7 times higher compared to men without diabetes (8.1%). As in women, obesity is therefore most strongly associated with diabetes in men. Figures across age groups for men are also similar to those found for women. Prevalence for men with diabetes (17.1%) is already 6.1 times higher in the younger age group of 18- to 29-year-olds compared to men without diabetes (2.8%). The highest prevalence of obesity in men with diabetes (36.5%) is found in the 30 to 59 age group; for men without diabetes, it is found in the 60 to 79 age group (8.9%). With age, the differences in prevalence become relatively smaller, which – as in women – coincides with a faster and relatively greater decrease in the prevalence of obesity in the group with diabetes compared to the group without diabetes.

Across all age groups, the prevalence of high blood pressure in men with diabetes is 79.0% and is 1.4 times higher than in men without diabetes (55.1%). With regard to high blood pressure, the picture is similar to that of women across all age groups. For men, too, the relative difference is highest in the 18 to 29 age group, with a 4.8 times higher prevalence (14.3%) in the group with diabetes compared to the group without diabetes (3.0%). With age, prevalence in the groups gradually equalises and the PR factor decreases to 1.2 in the age group 80 years and older. The highest prevalence of high blood pressure is reached in the age group 80 years and older (persons with diabetes: 88%, persons without diabetes: 72.6%).

The prevalence of heart failure and stroke are identical or slightly higher across all age groups with values between 20.2% and 8.0% for men with diabetes and 10.8% and 4.9% for men without diabetes compared to the figures for women. Progressively, for all age groups, the prevalence for men with diabetes is higher compared to men without diabetes. The highest prevalence in both study groups is again found in the age group 80 years and older.

In spite of a similar PR, men are significantly more likely to be affected by CHD. CHD prevalence across all age groups is 33.0% in men with diabetes and 1.8 times higher compared to men without diabetes (17.6%).

In contrast to the figures for CHD, the prevalence of depression is lower in men in both groups compared to women. For men with diabetes, the prevalence of depression across all age groups is 15.9%, 1.4 times higher than for men without diabetes (11.4%). The highest prevalence of depression (17.6%) is found in men with diabetes in the 30 to 59 age group and in men without diabetes in the 30 to 59 age group (11.6%) but there are only minor differences to the higher age groups.

## 4. Discussion

Based on the full sample of ambulatory claims data of SHI-accredited physicians, the current study assessed the prevalence of important comorbidities of diabetes compared to persons without diabetes. In line with a study based on data from the AOK Baden-Württemberg [18], which specifically analysed the case of type 2 diabetes and was based on a comparable methodology, this study also shows a higher prevalence for persons with diabetes for each disease. In contrast to the aforementioned study [18], the results in this study are based on data from all SHI-accredited physicians in Germany and therefore allow conclusions to be drawn that apply to all diabetes patients covered by SHI. The results highlight both the importance of diabetes as a frequent consequence of behavioural risk factors and the strong links of diabetes with other diseases, especially cardiovascular disease. The study design provides insights into the specific age- and sex-related factors of frequent concomitant diseases of diabetes. Compared to people without diabetes, women and men with diabetes are considerably more likely to be severe overweight and have elevated blood pressure, even at a younger age. As a result, the burden of disease due to cardiovascular diseases, but also depression, is greatly increased across all age groups, but especially in people under 60 years of age.

### 4.1 Prevalence of documented diabetes

To estimate the development of the documented prevalence, the diabetes prevalence figures derived from Germany-wide 2019 claims data were compared to the 2013 diabetes surveillance figures. The documented prevalence in the earlier study was calculated using the data set provided according to Germany’s Data Transparency Ordinance (DaTraV data), which in addition to the ambulatory claims data used here includes inpatient claims data of all patients covered by SHI [7]. Across all adult age groups the prevalence found in DaTraV data in 2013 was 11.2% for women and 12.6% for men [29]. In comparison with the rates this study found for 2019 (11.0% for women and 12.3% for men), the prevalence is slightly lower below the 2013 figures. The small difference of prevalence estimates derived from DaTraV data and the present analysis indicate that the documented prevalence has stabilised at a high level, an interpretation also corroborated by another analyses of SHI claims data [19]. A further indication that the documented prevalence has stagnated at a high level is found when comparing the 2015 figures for people with diabetes, which were based on the same data source and case definition [30]. While the 2015 study identified 6,955,865 people with diabetes, this study counted 7,068,249 persons with diabetes in 2019, in spite of excluding persons younger than 18, who are, however, rarely affected by diabetes.

If we limit the analysis in this study to the age range covered by the population representative DEGS1 survey (2008–2011, 18- to 79-year-olds), the documented prevalence for women was 8.5% and 10.5% for men. According to DEGS1, the prevalence of known diabetes in relation to persons covered by SHI is 7.8% for women and 7.2% for men, with gestational diabetes accounting for 1.2% of the population-wide prevalence in women [3]. Assuming there is strong correlation between the prevalence of known diabetes in claims data and the diagnosis prevalence found by DEGS1 collected by physician interviews, the comparison of prevalence figures by sex of DEGS1 with the results of this study indicate a considerable increase in documented diabetes between the years when data for DEGS1 were collected and 2019. With regard to the documented prevalence, an earlier analysis of the nationwide ambulatory claims data of people covered by SHI [30] already showed an increase across all age groups from 9.00% to 9.96% between 2010 and 2015, which means a relative increase of around 11% or, in absolute terms, of around 700,000. Much of this increase (around 8%, or roughly 500,000) occurred between 2010 and 2013. A comparison of the years 2015 and 2019 shows that the case numbers for documented diabetes mentioned above stagnated. The higher documented prevalence in 2019 compared to DEGS1 is therefore presumably partly owed to a strong increase in prevalence in the years up to 2015. A further decrease in the proportion of undiagnosed diabetes since 2010 could very well account for a part of this difference, as its decrease would simultaneously mean an increase in documented cases of diabetes. In DEGS1, the proportion of undiagnosed diabetes was still 1.2% for women and 2.9% for men, and was thus already considerably lower than in the previous German National Health Interview and Examination Survey 1998 (GNHIES98) [1]. Clarification of the time trend will be provided by future population-wide examination surveys conducted by the RKI. In principle, the guidelines for diabetes diagnostics and in particular the threshold values used for measured parameters, such as long-term blood glucose levels (HbA1c value), incorporate new findings, which could also have an influence on the development of prevalence over time [31].

### 4.2 Obesity and high blood pressure prevalence in diabetes patients

The strongest association with diabetes, both in women and men, is seen for obesity and high blood pressure. This result is consistent with the biological mechanisms described, according to which people with obesity are more likely to develop diabetes, whereby obesity and diabetes are likewise considerable risk factors for developing high blood pressure [32]. As shown in DEGS1, the population representative prevalence of obesity (Body Mass Index ≥ 30 kg/m^2^) for people with type 2 diabetes (aged 45 to 79) was 54.4% [33] and thus higher than the prevalence determined in this study: in the 30 to 59 age group, the prevalence of obesity was 46.1% for women and 36.6% for men; in the 60 to 79 age group, the figures stood at 36.5% for women and 31.1% for men.

For the total population of 18- to 79-year-olds, DEGS1 shows an increased prevalence of obesity in women, especially of more severe forms, which is in line with the results from this study [34]. The pronounced decrease in prevalence for persons with diabetes shown in this paper from the 60 to 79 age group cannot be confirmed with the DEGS1 data published, as here the total population is considered. However, more severe forms of obesity, which are also more strongly associated with diabetes [35], already begin to decrease in the 60 to 69 age group in DEGS1 [34]. As there are published study results showing that accounting data predominantly document severe forms of obesity [36] and that persons suffering obesity and diabetes have an increased mortality [37], the results presented here indicating a high prevalence in young age groups combined with a decline occurring early in life are epidemiologically highly plausible.

DEGS1 shows a prevalence of drug-treated or measured high blood pressure of 76.4% for 45- to 79-year-old type 2 diabetes patients [38]. Notably, both the values for individuals in the 65- to 79-year-old age group in DEGS1 (85.5% for women and 80.3% for men) and for the 60- to 79-year-old age group in this study (women 84.5%, men 83.8%) are similarly high. Thus, the documented prevalence of high blood pressure in persons with documented diabetes in this study is comparable to that of DEGS1 – a result that is supported by the high validity of documented billing diagnoses for high blood pressure [36, 39].

Unlike high blood pressure prevalence, the prevalence of obesity in people with diabetes did not decline between the RKI surveys [33, 40]. Since obesity already becomes apparent at a young age and its development and course can be strongly influenced by behavioural and settings-based factors, there is considerable potential for prevention here with regard to the burden of disease and premature mortality.

### 4.3 Cardiovascular disease prevalence in diabetes patients

For persons with type 2 diabetes 45- to 79-years-old, DEGS1 estimates the prevalence of at least one cardiovascular disease to be at 37.1% [33]. Comparing this value with available study data is difficult due to the more detailed presentation of analysis for individual diseases from the larger group of cardiovascular diseases chosen here. However, if one assumes, for the purpose of comparison, that heart failure develops on the basis of CHD and that stroke, which occurs in older age groups, also overlaps with CHD, an estimate based on CHD alone is possible. According to this assumption, the prevalence found by DEGS1 is higher than the prevalence documented in the claims data in the 30 to 59 and 60 to 79 age groups in women (6.2%, 19.1%) and men (13.7%, 33.7%) as part of this analysis. This confirms the result of a recent study [11], which, based on the same data and case definition, shows a moderately lower prevalence for the documented prevalence of CHD compared to DEGS1. The strong association between the documented and population representative prevalence for CHD is also supported by the fact that the sex-specific characteristics of a considerably higher population representative raw disease prevalence for women are also reflected by claims data prevalences [11, 41].

### 4.4 Depression prevalence in diabetes patients

Compared to the diseases discussed so far, depression and diabetes differ in their biological mechanisms, as well as their risk and influencing factors. Nevertheless, an analysis of diabetes surveillance shows that 19.1% of women and 12.3% of men with diabetes show depression symptoms in adulthood [42]. Between the groups with and without diabetes, the analysis, moreover, shows that the age-adjusted likelihood for a person to develop depression symptoms is twice as high for people with diabetes compared to people without the disease [42]. International findings corroborate this [10]. In accordance with these results, this analysis shows a higher prevalence of depression for the group with diabetes and for women. For women aged 18 years and older with diabetes, this analysis shows a documented prevalence of 26.9% and 15.9% for men. The considerably higher figures found in the documented diagnoses relative to those found in survey data are known and have been discussed in detail elsewhere [14]. The main reason for this discrepancy is likely to be the specific definition of depression in clinical interviews, which determines the condition at a level of detail not possible using solely claims data [14, 43].

### 4.5 Strengths and limitations of the study

This study is based on all the ambulatory diagnoses of patients covered by SHI using claims data from SHI-accredited physicians. This avoids a distortion of the calculated Germany-wide documented prevalence that could result from different compositions regarding age structure and other risk factors among members of individual health insurance funds or SHI-accredited physician associations.

Compared to survey data, the inclusion of all age groups is a particular strength of claims data and thus of the study presented here. In particular, the old and very old were not included in the previous nationwide survey data, where the age range was limited to a maximum of 79. In addition, claims data are routinely collected regardless of a patient’s willingness to participate. Consequently, the data covers large swathes of the population. Ultimately, the scope of information made available through claims data also allows for deeply stratified analyses by age, region and sex. Overall, the fast availability of SHI-accredited physician claims data within less than one year is advantageous. These data thus make it possible to show changes in morbidity quickly. A fundamental disadvantage of SHI claims data is that patients with private health insurance are not included and that services provided outside the statutory claims system are not documented. Although the majority of the German population is covered by SHI, it is estimated that information about the illness history of 12.2% of the population is not recorded in these data, and they are therefore not representative of the population [44].

Especially in comparison with the population representative DEGS1 study, the prevalences calculated in this study for 2019 correspond well with the epidemiological results by sex and age group. In particular regarding diseases where billing diagnoses and clinical diagnoses are known to show a strong correlation, such as diabetes, high blood pressure and cardiovascular diseases, the study results are robust. However, compared to the prevalences found in examination surveys such as DEGS1, risk factors such as being overweight and obesity appear to be under-coded or not recorded. The documented prevalence of depression calculated in our study, which is considerably higher compared to the prevalence found by DEGS1, is difficult to classify. Studies show that depression, in particular, is coded considerably more often in claims data than in clinical diagnosis data [14, 43, 45]. This study does not operationalise patients’ social situation as the corresponding indicators often used, such as income, occupational status or educational status, are not present in the data. For this reason, the prevalence ratios presented here are not adjusted for differences in educational attainment between people with and without diabetes. In particular, as results show diabetes prevalence reflects social inequalities [46], the prevalence ratios presented here are skewed to a degree that depends on the unknown distribution of social situation indicators between persons with and without diabetes.

In general, it must be assumed that risk factors and concomitant diseases are also coded more frequently in persons with a documented chronic disease such as diabetes [36, 39, 47]. To mitigate this effect, in this analysis the persons with diabetes were compared to a control group of persons with an appointment at a SHI-accredited physician in at least two quarters.

In contrast to the data from SHI providers, the data in this study do not include inpatient diagnoses, drug prescriptions or, in particular, persons without ambulatory encounter. Due to the lack of people with SHI without ambulatory encounter, prevalence cannot be calculated on the basis of the data alone, as the total population of people with SHI cannot be determined directly. This study addressed this limitation by using the official member statistics of SHI providers – called the KM 6 statistics – to estimate the total SHI population. For the other limitations, i.e. absence of inpatient diagnoses and drug prescriptions, the comparison of the study’s documented prevalence with the results of the literature considered in this article shows that for common chronic diseases, the ambulatory SHI-accredited physician care of all persons covered by SHI captures the disease situation well.

## 5. Conclusion

Using current and Germany-wide ambulatory claims data, this study underscores that, on the whole, persons with diabetes, but especially those at younger adult age, have a considerably increased disease burden due to severe overweight, elevated blood pressure and cardiovascular disease. The claims data of all ambulatory services provided by SHI-accredited physicians are suited to continuously assess diseases of high public health relevance. In particular, diabetes surveillance at the RKI could benefit from a regular assessment of diabetes prevalence and diabetes comorbidities as documented in claims data. If repeated, the chosen study approach would also enable estimates of changes in the comorbidity burden in a comparison of persons with and without diabetes.

Lastly, the ongoing COVID-19 pandemic underlines the importance of systematically monitoring and assessing the development of diabetes and diabetes comorbidities. Analyses show that persons with diabetes, obesity, cardiovascular as well as other chronic diseases also suffer greater complications when they develop COVID-19, such as hospital admission, ventilation or death, regardless of age and are more likely to die from the disease [48]. Similar effects have also been documented for other viral infections such as seasonal influenza [49]. An improved health situation and care of the population would likely also lead to a decrease in the number of severe courses of the disease [50].

## Key statements

- Women and men with diabetes show a higher prevalence for all studied comorbidities and across all age groups compared to women and men without diabetes.
- In comparison over time, the documented prevalence of diabetes in 2019 stagnates at a high level.
- The highest relative difference in prevalence between people with and without diabetes in women and men is found for obesity.
- With the exception of depression, the documented prevalence of the examined diseases shows good agreement with population representative prevalence.
- Claims data of all ambulatory statutory health insurance physicians are well suited for the regular analysis of diabetes comorbidity in diabetes surveillance.

## Figures and Tables

**Table 1:** Features of the two groups analysed (case and control group) Source: Germany-wide claims data from SHI-accredited physicians for adults covered by statutory health insurance, own calculations

	Case group with diabetes	Control group without diabetes
Number of persons	7,068,249	7,068,249
Proportion of women in %	49.79	49.79
Average age (SD)	68.99 (13.71)	68.93 (13.84)
Treatment cases^1^ per person and year (mean value)	14.21	10.98
Services^2^ per person and year (mean value)	126.75	83.57
Value of services in euros (mean value)	1,147.98	815.50

SD = standard deviation

^1^ Treatment cases are defined in § 21 Para. 1 of the Bundesmantelvertrag-Ärzte (BMV-Ä) as treatment of the same insured person by the same medical practice in a calendar quarter at the expense of the same health insurance fund [28].

^2^ This indicator records the number of invoiced fee schedule items for individual medical services, such as home visits or specific diagnostic and therapeutic services, but also invoiced fee schedule items that represent flat rates for service complexes, such as primary care or specialist care.
